# Large-Area and Patternable Nano-Dot Array from Electrolysis of ITO Film for Surface-Enhanced Raman Spectroscopy

**DOI:** 10.1186/s11671-019-3239-9

**Published:** 2020-01-13

**Authors:** Han Lu, Gengxin Han, Jieping Cao, Mingliang Jin, Qilin Ma, Eser Metin Akinoglu, Xin Wang, Li Nian, Guofu Zhou, Lingling Shui

**Affiliations:** 1 Guangdong Provincial Key Laboratory of Optical Information Materials and Technology, South China Academy of Advanced Optoelectronics, South China Normal University, Guangzhou, 510006 China; 2 International Academy of Optoelectronics at Zhaoqing, South China Normal University, Zhaoqing, 526238 China; 3 School of Information and Optoelectronic Science and Engineering, South China Normal University, Guangzhou, 510006 China

**Keywords:** Nano-dot, SERS, Electrolysis, Array, High-throughput sensing

## Abstract

Fabrication of large-area devices with patternable nanostructures is important for practical applications in optical or electrical devices. In this work, we describe an easy and environment-friendly method for preparing large-area nano-dot (ND) arrays via the electrolytic reaction of a metal oxide film. NDs with various size and morphology can be obtained by adjusting the applied voltage, electrolysis time, and the film thickness of the indium tin oxide (ITO) layer. High-density NDs with size of 50–60 nm can be obtained by electrolysis of a 25-nm-thick ITO film at 150 V for 1.5 min under a water droplet medium, which have been applied for surface-enhanced Raman spectroscopy (SERS) after depositing a thin layer of silver. The SERS substrate with optimized ND structure exhibits sensitive detection of Rhodamine 6G (R6G) with detection limit down to 5 × 10^-12^ M. The enhancement factors (EFs) of 1.12 × 10^6^ and 6.79 × 10^5^ have been achieved for characterization of 4-methylbenzenethiol (4-MBT) and R6G, respectively. With an additional photolithographic step, multiple areas of ND arrays can be created on one substrate, enabling simultaneous detection of various samples containing different molecules at once experiment. Such a method is quick, easy, patternable, and environment-friendly, being suitable for on-site quick and synchronous determination of various molecules for applications in point-of-care, environmental monitoring, and airport security fields.

## Introduction

Surface-enhanced Raman scattering (SERS) was observed a few decades ago from the roughed surface of silver electrode [1]. It has been widely investigated not only to understand the mechanism of SERS but also to achieve practical applications. Two classical series of SERS substrates have been developed, the self-assembled colloidal materials and the nanofabricated structures [2]. Nanoparticles of coinage metals like Ag, Au, and Cu have been synthesized for SERS studies [3, 4]. The nanoparticle systems are easy-to-use, however lack of repeatability and reproducibility with relatively low sensitivity [5, 6]. Nanostructures fabricated by e-beam lithography [7, 8], laser interference lithography [9, 10], focused ion beam lithography [11], nanosphere lithography [12], and nanoimprint lithography [13] have shown high signal enhancement with excellent repeatability. However, these nanofabrication technologies require expensive equipment and restrict environment like ultraclean room; and the fabrication process is slow, as well.

Since SERS can be directly used for molecular sensing and identification in aqueous medium without interruption of water, it has been widely applied for small and bimolecular sensing [14–16]. For better and wider applications, easy and quick fabrication of SERS microarray substrate is still highly required for simultaneous detection of various molecules, especially for applications in point-of-care technology (POCT) and safety monitoring. Both colloidal particle assembly and nanofabrication technologies involve various types of chemicals or high energy consumption, for instance, special chemicals or gasses for particle synthesis and dry etch processes, respectively, and high energy consumption for sophisticated layer-by-layer design and deposition. Various environment unfriendly pollution are produced during the processes, such as organic, acid, base, heavy metal ions, and toxic etchant gas.

ITO films can be prepared via standard metal deposition technology and widely utilized in lab and industry as conductive substrate according to its transparency and low cost. Gao et al. have reported that ITO film could be transformed to indium (In) dots under cathodic polarization in NaOH solution [17].

In this work, we propose and verify a simpler, quicker, and greener technology by creating NDs on glass surface via direct electrolysis of ITO film in water in one step. With an additional photolithographic process, ND arrays can be created with patterned multiple separated areas and thus achieving simultaneous determination of multiple samples containing various types of molecules on one substrate. The electrolysis takes place under mild conditions at low voltage in water environment/medium.

## Materials and Methods

### Materials and Reagents

ITO glass (1.1-mm thick) was purchased from Luoyang Longqian Glass Co., Ltd. (Henan, China), with ITO thickness of 25, 50, 100, and 200 nm corresponding to the square resistance of 93.52, 31.05, 15.86, and 6.97 Ω/sq, respectively. Fluorine-doped tin oxide (FTO) glass (2.2-mm thick) was purchased from Yaoke Photoelectric Co., Ltd. (Jiangsu, China), with FTO thickness of 400 nm and square resistance of 10.85 Ω/sq. Deionized (DI) water (18.25 MΩ cm at 25 °C) was prepared using a Milli-Q Plus water purification system (Sichuan Wortel Water Treatment Equipment Co., Ltd., Sichuan, China). Ethanol (Damao Chemical Reagent Factory, Tianjin, China) and acetone (Zhiyuan Chemical Reagent Co., Ltd., Tianjin, China) were used to clean ITO glass. Photoresist SUN-120P was purchased from Suntific Microelectronic Materials Co., Ltd. (Shandong, China) for patterning ITO. 4-Methylbenzenethiol (4-MBT, 98%), sodium 2-mercaptoethanesulfonate (MESNa, ≥ 98.0%) and dopamine hydrochloride were all purchased from Sigma-Aldrich (St. Louis, MO, USA). Rhodamine 6G (R6G, 98.5%) was purchased from J&K Scientific (Beijing, China). Potassium hydroxide (KOH, GR 95%) and melamine (99%) were purchased from Aladdin (Shanghai, China). D-(+)-Glucose (99%) was purchased from Alfa Aesar (Shanghai, China). Methylene blue (AR), urea (AR, ≥ 99.0%) and phosphoric acid (AR, 85%) were purchased from Damao Chemical Reagent Factory (Tianjing, China). Formaldehyde solution (AR, 37–40%), sodium dihydrogen phosphate dehydrate (AR, ≥ 99.0%), and disodium hydrogen phosphate dodecahydrate (AR, ≥ 99.0%) were purchased from Guangzhou Chemical Reagent Factory (Guangzhou, China). All chemicals were used as received.

### Electrolysis of ITO Film in Water

**Fig. 1:**
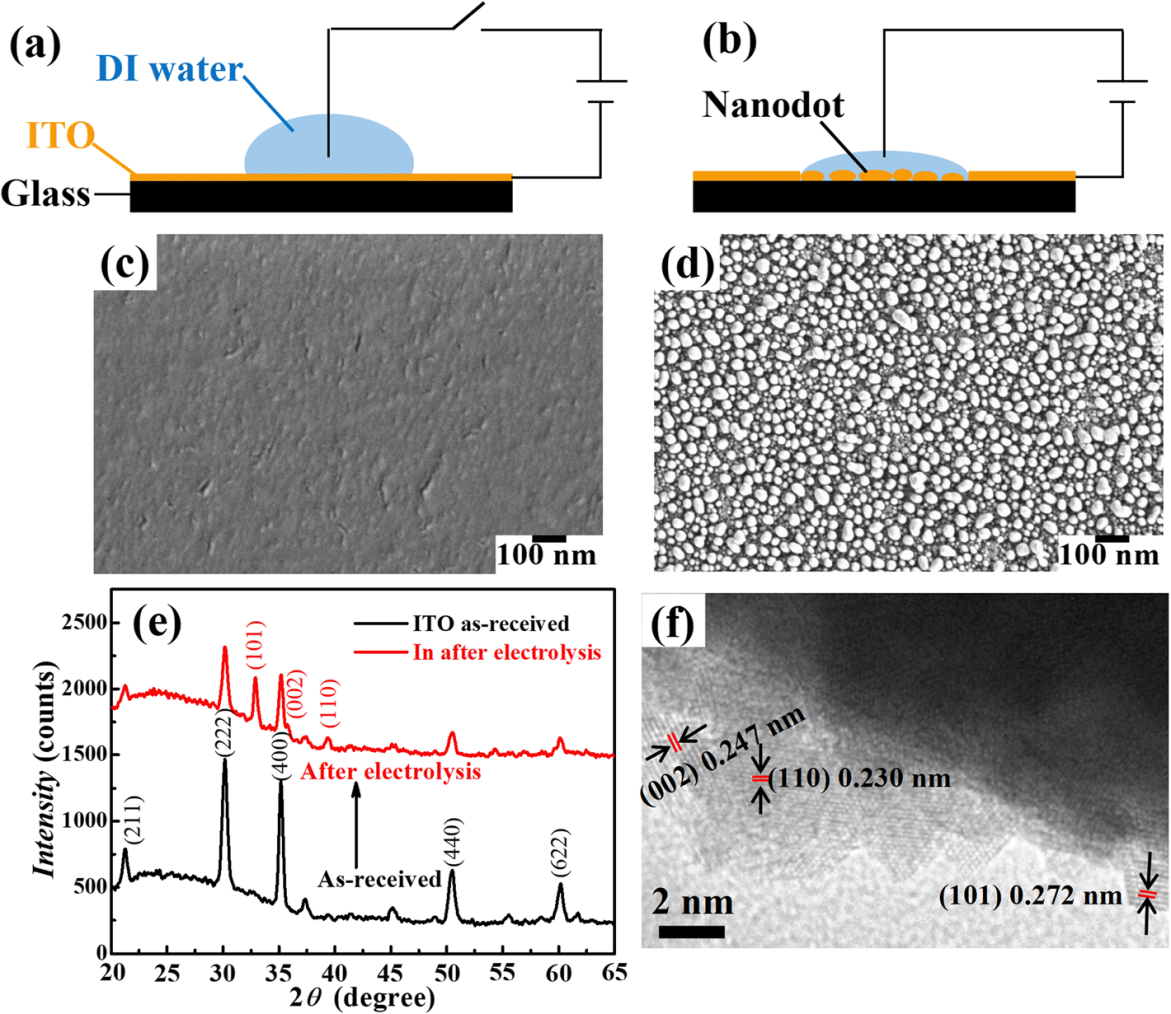
Schematic of the In NDs formation from electrolysis of ITO film. **a** Initial state of a water droplet standing on an ITO film without applying a voltage. **b** Formation of NDs on the area covered with water when a voltage is applied. **c** SEM image of the intact ITO film surface corresponding to **a**. **d** SEM image of the formed NDs corresponding to **b** (applied voltage of 150 V for 1.5 min at ITO film thickness of 25 nm). **e** XRD spectra of the ITO film before and after electrolysis. **f** TEM image of the obtained In NDs

An ITO glass substrate was first cut and cleaned sequentially in acetone, ethanol, and DI water, with each process lasting for 15 min, and then thoroughly rinsed with DI water and dried using nitrogen blowing. Figure 1 shows the schematic of the fabrication process. A droplet of DI water was dripped onto the ITO surface as the electrolysis medium. A wolfram (W) wire was inserted in the droplet and connected to the positive electrode, and ITO film was connected to the negative electrode of a power supplier (PSW800-1.44, GWINSTEK, Taiwan, China), as drawn in Fig. 1a. When a voltage was applied across the water, ITO was electrolyzed to form In NDs within the water medium area (Fig. 1b).

Moreover, an ITO film can be easily patterned to various areas in micrometer to centimeter range. An ITO film can also be patterned to an array with various isolated areas consisting NDs to achieve multiple functional areas on one substrate. This is very important for high-throughput/multiple detection devices. In order to form patterned ND arrays, 3.5 × 3.5 cm^2^ ITO-glass surface was patterned to separate square areas via a photolithography process. Afterwards, a grid-patterned ITO substrate and a flat ITO substrate were bonded face-to-face to form an in-parallel device sealed with 200-μm-thick press adhesive frame and filled with DI water. The flat and patterned ITOs were connected to the positive and negative electrodes of a power supplier, respectively. This method was highly suitable for preparation of large-area and patterned ND arrays.

### SERS Measurements

By tuning the applied voltage, electrolysis time, and ITO film thickness, the NDs’ size and density could be varied. Formed NDs were typically tens to hundreds of nanometers arranged closely, forming nanogaps. A SERS substrate was then obtained by depositing a thin layer of Ag (PD400, Wuhan PDVACUUM Technologies Co., Ltd., Wuhan, China).

To characterize the sensitivity of such a SERS substrate, Raman spectra were measured using a Renishaw inVia Raman Microscope (Renishaw 42 K846, Renishaw Co., Ltd., UK). A SERS substrate was immersed into the analyte solution for 1 h and then thoroughly rinsed for 1 min using the corresponding solvent and blow dried with pure nitrogen gas. Dried SERS substrate was then placed on the stage of the Raman microscope for measurements. A 532 nm laser with a power of ~ 0.14 mW was focused on the sample through a × 50 objective lens (numerical aperture, NA = 0.5, Leica). The diameter of the laser spot on substrate was 1.30 μm. The elastically scattered laser excitation was removed with an edge filter. Each Raman spectrum was collected with 10 s accumulation time.

Moreover, to evaluate the versatility and quick detection, nine probe molecules of 4-MBT, R6G, dopamine hydrochloride, urea, formaldehyde, methylene blue, MESNa, d-(+)-Glucose, melamine were chosen to characterize the prepared SERS substrate for simultaneous determination of multiple molecules. 4-MBT and methylene blue were dissolved in ethanol. MESNa was dissolved in DI water with pH of 2.7 tuned by the phosphate buffer solution. d-(+)-Glucose solution was prepared by using a phosphate buffer solution with pH of 7.5. All the rest sample solutions were prepared by dissolved in DI water. For each measurement, a droplet of 3.0 μL prepared solution was quickly dripped on the substrate, and the Raman spectrum was recorded immediately. The analyte concentrations were 10^-4^, 5 × 10^-11^, 10^-3^, 0.5, 10^-3^, 10^-5^, 10^-2^, 1, and 10^-2^ M for 4-MBT ethanol solution, R6G aqueous solution, dopamine hydrochloride solution, urea aqueous solution, formaldehyde solution, methylene blue ethanol solution, MESNa solution, d-(+)-glucose solution, and melamine aqueous solution, respectively. All measurements were carried out immediately using the Raman instrument (Finder Insight, Zolix Instruments Co., Ltd., Beijing, China) with an excitation laser of 532 nm wavelength and 2.5 mW power. The 10-μm diameter laser beam was focused on the sample through a × 50 objective (NA = 0.55). Each Raman spectrum was collected for 0.3 s/time, with 10 times accumulation.

### Other Characterizations

The morphologies of fabricated substrate were characterized using a field emission-scanning electron microscopy (FE-SEM) (ZEISS-Ultra 55, Carl Zeiss AG, Germany) at an acceleration voltage of 5 kV. Transmission electron microscopy (TEM) measurements were carried out using a JEM-2100 microscopy (JEM-2100HR, JEOL, Japan). The nanodots (nanoparticles) were scraped and dispersed in DI water. Chemical composition was analyzed using an energy dispersive spectroscope (EDS) equipped in the FE-SEM and an X-ray diffraction (XRD) (X’Pert PRO, PANalytical, The Netherlands) equipped with a Cu Ká radiation source, at a scan rate of 0.04°/s, and the diffraction angle (2*θ*) from 20 to 65°. The absorption spectrum was recorded in the wavelength range of 440–650 nm by using a spectrometer (USB 2000+, Ocean Optics, USA).

## Results and Discussion

### Formation of Nanodots by Electrolysis of ITO Film in Water Medium

ITO films as transparent conductive substrates have been widely applied in optoelectronic devices such as light emission device (LED) [18], display [19], and solar cell [20]. In general, ITO corrosion is detrimental for electronic device applications. Here, on the other hand, we make use of the ITO corrosion induced by electrolysis reaction to form closely packed NDs and used for SERS application. Schematic of the electrolysis induced ND formation is shown in Fig. 1a, b. ITO film surface was flat and transparent before electrolysis (Fig. 1c). In general, ITO is a composed of In_2_O_3_ and SnO_2_ in various proportion. EDS was carried out to characterize the composition of ITO, as shown in Additional file 1: Figure S1. After a period of electrolysis reaction, the ITO surface became translucent and yellow after drying. Characterized by SEM, we found that closely packed NDs on the glass surface were formed on the glass surface (Fig. 1d). XRD measurement in Fig. 1e shows that three new peaks appeared after electrolysis, which corresponds to (101), (002), and (110) crystal planes of In element. However, the peaks of ITO became lower. TEM image in Fig. 1f confirms that the formed NDs are of In material.

ITO film is a metal oxide material with morphologies of both crystalline and amorphous, with typically nanoscale surface roughness [21]. It has been reported that ITO can be corroded by NaOH to form In nanoparticles [17]. When a voltage is applied across the ITO film, electrons are transferred between cathode and anode. Therefore, the electrochemical reactions on cathode and anode can be described by Eqs. (1) and (2):

1 $$ \mathrm{Cathode}:{\mathrm{In}}_2{\mathrm{O}}_3+3{\mathrm{H}}_2\mathrm{O}+6{\mathrm{e}}^{-}\to 2\mathrm{In}+6{\mathrm{O}\mathrm{H}}^{-} $$

2 $$ \mathrm{Anode}:4{\mathrm{O}\mathrm{H}}^{-}\to {\mathrm{O}}_2+2{\mathrm{H}}_2\mathrm{O}+4{\mathrm{e}}^{-} $$

**Fig. 2:**
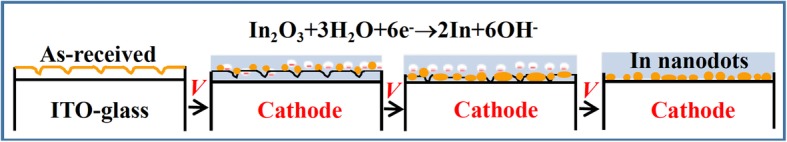
Schematic illustration of the formation process of In NDs from electrolysis of ITO film

Therefore, the ND formation process can be illustrated in Fig. 2. In the beginning, the electrolysis reaction is homogeneous over the ITO surface. However, the ITO film is not perfectly homogeneous with film thickness variation existed according to normal ITO fabrication process, especially for amorphous ITO films [22]. Therefore, with time evolution, the thinner area will be consumed faster to form defects according to the higher electric field strength and smaller thickness. After reaching the saturation concentration in water, reduced In atoms start to accumulate to form NDs on surface. During the electrolysis, obvious transparency and color change could be observed after a period of time. According to the interfacial tension effect during a dewetting process, a large quantity of NDs was formed on the surface. Such a method, without any requirements for specific treatment and chemicals, is realized in water medium with an applied voltage and thus can be considered as an environment friendly technology.

### Electrolysis Parameters: Reaction Time, Applied Voltage, and ITO Film Thickness

**Fig. 3:**
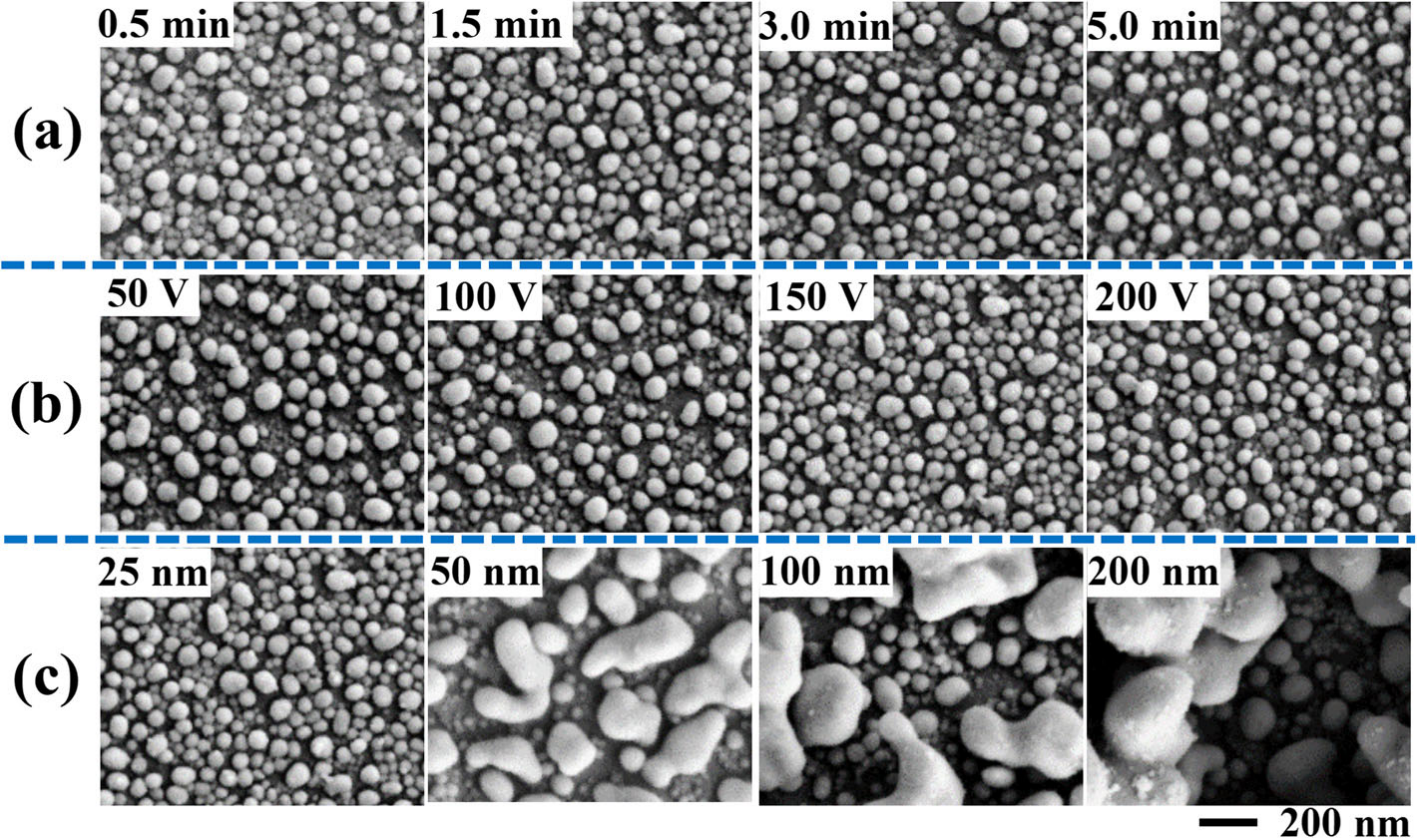
Fabrication of NDs under various experimental conditions by varying **a** reaction time, at a constant applied voltage of 150 V and ITO film thickness of 25 nm, **b** applied voltage, at constant ITO film thickness of 25 nm and reaction time of 1.5 min, and **c** ITO film thickness, at constant applied voltage of 150 V and reaction time of 1.5 min

The NDs’ size and density are related to the ITO film thickness and the reaction kinetics [23]. In this work, the effective factors of reaction time, applied voltage, and ITO film thickness were all investigated to find out the ND formation process. In this way, experimental parameters could be optimized to prepare SERS substrate with high sensitivity. Figure 3a shows the SEM images of obtained NDs on the glass (25-nm-thick ITO) at an applied voltage of 150 V at different reaction time. It obviously shows the sequential formation process from a continuous ITO film (Fig. 1c) to small NDs embedded in the film, rough NDs, smooth NDs, and standing and separated NDs, as demonstrated in the images in Fig. 3a from left to right, respectively. This gradual change in morphology and size can be understood by the electrolysis reaction of the ITO film and the diffusion-controlled ND formation process.

Initially, the ITO film started to react on the film surface. Typically, the electric field strength across the thinner area is higher; thus, the initial reaction speed is higher. Consequently, defects would form on the continuous film from the thin points (area), in which the produced In atoms accumulated to form NDs. At this stage, formed small NDs were still in the defect areas, being surrounded by the ITO film. With the increase of reaction time, most of ITO material was reduced to form In NDs on the surface. When the reaction time reached 0.5 min, large amount of NDs were formed, being embedded in the ITO film. When the reaction time increased to 1.5 min, the NDs’ size and density increased, and the gap between NDs decreased. Further increasing the reaction time to 3.0 and 5.0 min, obtained NDs became larger and rounder, and the gap among NDs increased as well. Electrical measurements showed that the ND areas were not electrically conductive. This means that isolated NDs were obtained without continuous interconnection. From the SEM images in Fig. 3a, we could observe that, at the reaction time of 1.5 min, the formed NDs have relatively uniform size and arranged closely. Smaller gap usually means stronger electromagnetic enhancement. Thus, 1.5 min was selected for preparing the SERS samples for further experiments.

Afterwards, we investigated the effect of applied voltage on the size and density of formed NDs. An ITO-glass with a 25-nm-thick ITO has been chosen for this experiment, and an electrolysis reaction was carried out at different applied voltage for 1.5 min. As shown in Fig. 3b, the density of formed NDs increased with applied voltage. At low voltage of 50 and 100 V, the quantity of formed NDs was low; and thus, in the same area, its density was low, being obviously separated to each other. When the applied voltage was increased to 150 and 200 V, more NDs were formed, showing closely arranged patterns. Uniform size and high density are essential to acquire reproducible Raman spectra with high sensitivity. Thus, the optimal applied voltage for SERS substrate preparation was set at 150 V.

Figure 3c shows the SEM images NDs formed from 25, 50, 100, and 200 nm ITO films by electrolysis reaction at 150 V for 1.5 min. In the case of 25 nm ITO film, higher density and more uniform NDs were observed compared with other three ITO films with thickness of 50, 100, and 200 nm. As reported, the surface roughness and resistivity of conductive substrates affect its crystallinity [22]. Typically, the surface roughness increases with film thickness. More uniform NDs from thinner ITO film were attributed to the more flat surfaces with fewer defects. Therefore, the thinnest ITO film exhibited the lowest roughness, resulting in the most uniform NDs. On the other hand, the resistivity of ITO films would influence the initial In ND formation process. The square resistance was 93.52, 31.05, 15.86, and 6.97 Ω/sq for the ITO film thickness of 25, 50, 100, and 200 nm, respectively. This means, at the same applied voltage, low electric current was obtained across the thin ITO film. As a result, slow and mild reaction was achieved on thin film. This is consistent with the experimental result that more uniform and higher density NDs were formed from the thinner ITO films. According to these results, the experimental parameters for preparing the NDs for SERS application were selected to be of ITO film thickness of 25 nm, applied voltage of 150 V, and electrolysis reaction time of 1.5 min. Moreover, the FTO film has also been applied for electrolysis. As shown in Additional file 1: Figure S2, micro- and nano-particles formed after electrolysis. This may suggest that such an electrolysis reaction is also applicable for other metal oxide films at various conditions and potential for other applications.

### SERS Characterization

To evaluate SERS effect of the fabricated substrate with high density NDs, 4-MBT was selected as the probe molecule because of its small amount of well-characterized peaks and large Raman cross-section [24]. A thin layer of Ag was deposited on the obtained substrate (prepared under the optimized conditions as mentioned above) with high density NDs. SEM images in Additional file 1: Figure S3a–c show the morphologies of Ag covered NDs, at Ag layer thickness of 30, 77, and 160 nm, respectively. By varying the Ag layer thickness, the gaps between NDs decreased. The highest average Raman intensity was obtained at Ag thickness of 77 nm (Additional file 1: Figure S3d).

**Fig. 4:**
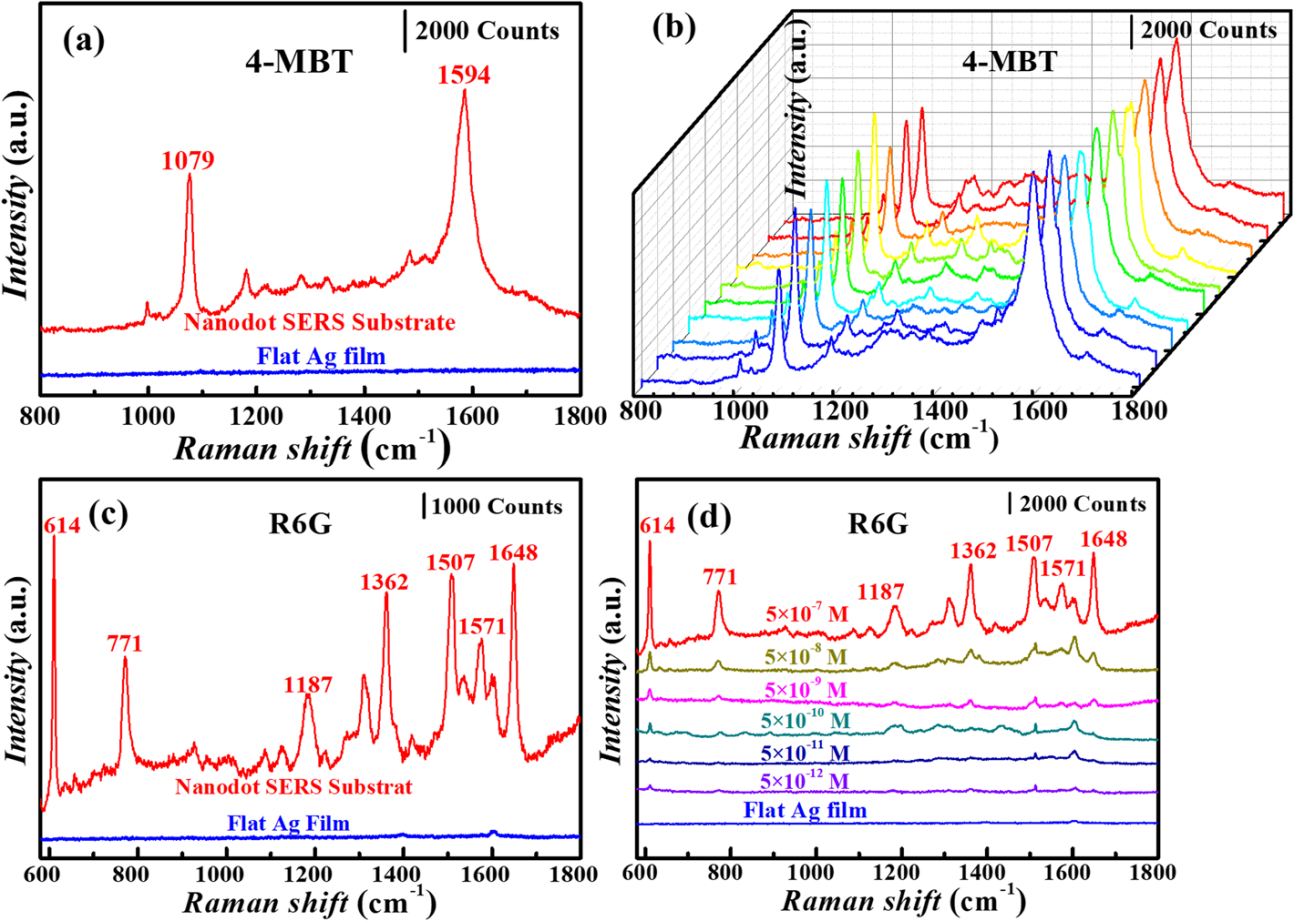
**a** Raman spectra of 10^-4^ M 4-MBT on a SERS substrate with NDs prepared under the optimized conditions. **b** 10 Raman spectra of 10^-4^ M 4-MBT collected on a SERS substrate by randomly moving the substrate on the stage of the Raman instrument. **c** Raman spectra of 5 × 10^-7^ M R6G on a prepared SERS substrate. **d** Raman spectra of R6G with concentrations of 5 × 10^-12^, 5 × 10^-11^, 5 × 10^-10^, 5 × 10^-9^, 5 × 10^-8^, and 5 × 10^-7^ M on SERS substrates. The reference substrate was prepared by depositing 77 nm Ag on a bare ITO (25 nm) film.

Figure 4a, b shows the detailed SERS characterization on the substrate with In NDs prepared under the optimized conditions and covered with 77 nm deposited Ag. The reference sample was prepared by directly depositing 77 nm Ag film onto an ITO (25 nm) glass. Raman signal was enhanced significantly on the ND SERS substrate compared to the reference substrate. The two major characteristic peaks for 4-MBT molecules at 1079 and 1594 cm^-1^ were clearly observed on the ND SERS substrate. The 1079 cm^-1^ peak represents a combination of the phenyl ring-breathing mode, C–H in-plane bending and C–S stretching. The peak at 1594 cm^-1^ can be ascribed to phenyl stretching motion (8a vibrational mode) [25].

To investigate the relative homogeneity over a large area of the prepared ND SERS substrate, 10 measurements were carried out on the same substrate by randomly moving the sample on the stage of the Raman instrument. Figure 4b shows the measured Raman spectra, suggesting relatively consistent signal intensities for each characteristic peak of the 4-MBT. The relative standard deviation (RSD) of the Raman intensity at 1594 cm^-1^ was about 4.1%, indicating the high signal reproducibility of the SERS substrate prepared via this proposed process.

Moreover, R6G has also been selected to demonstrate the SERS substrate’s reliability and sensitivity. Figure 4c shows the Raman spectra measured on an as-prepared ND SERS substrate and a reference Ag film. The characteristic peaks of R6G were observed at 614, 771, 1187, 1362, 1507, 1571, and 1648 cm^-1^. The peaks at 614, 771, and 1187 cm^-1^ are ascribed to C–C–C ring in-plane bending, C–H out-of-plane bending, and C–O–C stretching vibrations, respectively. And the peaks at 1362, 1507, 1571, and 1648 cm^-1^ are associated with aromatic C–C stretching vibrations [26].

Figure 4d exhibits the Raman spectra on an as-prepared ND SERS substrate in R6G aqueous solution with concentrations ranging from 5 × 10^-12^ to 5 × 10^-7^ M. Raman intensities of R6G decreased obviously with the decrease of R6G concentration. The characteristic peaks of R6G could still be identified clearly even at the R6G concentration as low as 5 × 10^-12^ M, indicating the high sensitivity of the fabricated ND SERS substrate. To quantitatively characterize the SERS effect of prepared ND substrate, we have calculated the enhancement factor (EF). The Raman spectra of pure powders of 4-MBT and R6G (Additional file 1: Figure S4), and the detailed information of EF calculation (Additional file 1: Figure S5) are presented in the supplementary information. EFs for 4-MBT and R6G were calculated to be 1.12 × 10^6^ and 6.79 × 10^5^ at their characteristic Raman peaks of 1079 and 1648 cm^-1^, respectively. Moreover, molecules with smaller Raman cross-section of MESNa [27] have also been used for SERS measurement, as shown in Additional file 1: Figure S6, demonstrating the reliable SERS effect.

In general, SERS enhancement can be contributed from electromagnetic (EM) and chemical (CM) effects [28]. Additional file 1: Figure S7 shows the absorption spectrum of ND substrate coated with 77 nm Ag film. The reference substrate was prepared by depositing 77 nm Ag on a bare ITO (25 nm) film. The NDs substrate was fabricated under the optimized experimental parameters of ITO film thickness of 25 nm, applied voltage of 150 V, and electrolysis reaction time of 1.5 min. When the excitation wavelength is the same as or close to the peak of surface plasmon resonance (SPR), the electromagnetic plasmonic coupling will take place and induce strong SERS enhancement [29]. The SPR peak of the NDs SERS substrate is at ~ 453 nm (Additional file 1: Figure S7), which is close to the excitation wavelength of 532 nm used in our experiment; therefore, the SERS enhancement is mainly resulted from the electromagnetic enhancement according to the “hotspots” from the inter-gaps between NDs. To further investigate the EM enhancement, finite-difference time-domain (FDTD) simulation was carried out to study the electric field in the inter-gaps of NDs. The results of relative total electric field are shown in Additional file 1: Figure S8. Simulation results show that the electric filed enhancement mainly occurs at the gaps between NDs. The maximum factor of 3.0 represents a field enhancement |E|^2^ of 10^3^ corresponds to a EF of 10^6^, which is in good agreement with the experimental results (1.12 × 10^6^ at 1079 cm^-1^ for 4-MBT and 6.79 × 10^5^ at 1648 cm^-1^ for R6G).

Moreover, the semiconductor materials (In_2_O_3_, SnO_2_, TiO_2_) have been reported to enhance SERS signal by charge transfer between the molecules and materials (e.g., R6G and transition metal oxides, 4-mercaptobenzoic (4-MBA)/4-nitrobenzenethiol (4-NBT), and SnO_2_), which is related to CM [28, 30–33]. ITO is a composed of In_2_O_3_ and SnO_2_ in various proportion. After ITO electrolysis in water medium, the In NDs are formed due to the electrochemical reduction reaction; and at the same time, the peaks of ITO could still be observed obviously as shown in Fig. 1e. To investigate how much the CM enhancement from In_2_O_3_/SnO_2_-to-molecule charge transfer contributed to obtained SERS effect, the Raman spectra of 10^-4^ M 4-MBT and 5 × 10^-7^ M R6G on the ITO glass, NDs’ substrate after ITO electrolysis, ITO glass coated with 77 nm Ag film, and NDs’ substrate coated with 77 nm Ag film were measured, respectively (Additional file 1: Figure S9). The NDs’ substrate was fabricated under the optimized experimental parameters of ITO film thickness of 25 nm, applied voltage of 150 V, and electrolysis reaction time of 1.5 min. The characteristic Raman peaks of 4-MBT and R6G were difficult to distinguish on the ITO glass, NDs substrate after ITO electrolysis, and ITO glass coated with Ag film; however, the obvious Raman peaks of 4-MBT and R6G were observed on NDs substrate coated with Ag film. Thus, the CM enhancement from In_2_O_3_/SnO_2_-to-molecule charge transfer is considered weak, being negligible comparing to EM enhancement. The highly enhanced SERS effect is mainly contributed from the electromagnetic enhancement between the inter-gaps between In NDs.

### Patterned ND Arrays for Simultaneous SERS Characterization of Various Samples on One Substrate

**Fig. 5:**
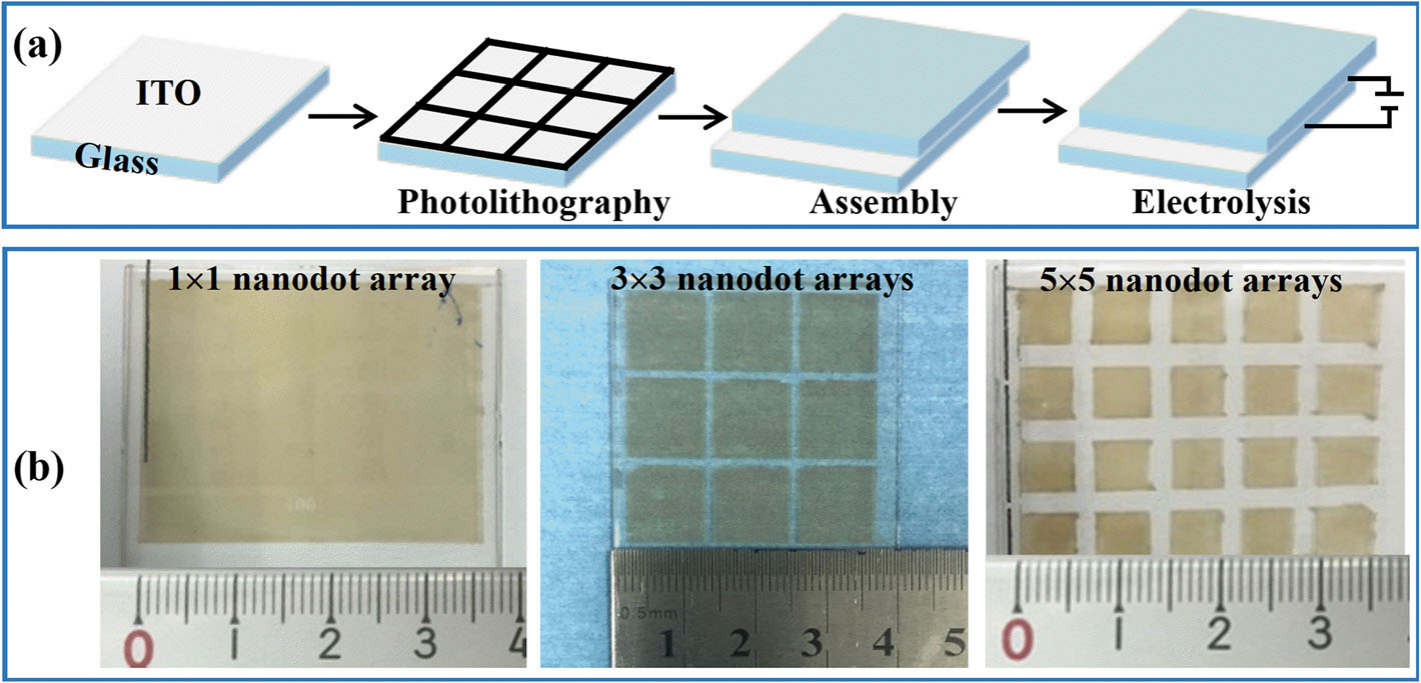
**a** Schematic of patterning an ITO film to an array with multiple isolated areas containing NDs. **b** Images of the 1 × 1, 3 × 3, and 5 × 5 SERS arrays patterned from three 3.5 × 3.5 cm^2^ ITO-glass substrates

As demonstrated in the previous session, this method could easily create the NDs by a simple and quick electrolysis reaction on nanoscale ITO film. Moreover, an ITO film can be patterned by partially protecting or segmenting the film, thus forming arrays on one substrate, as schematically demonstrated in Fig. 5a. Three 3.5 × 3.5 cm^2^ substrates have been patterned to 1 × 1, 3 × 3, and 5 × 5 SERS areas, as demonstrated in Fig. 5b. Since the ITO area for electrolysis reaction was enhanced compared to the droplet electrolysis, the total charge number increases during electrolysis, inducing the increase in current and decrease in resistance. According to the current limit of the power supplier of 1.512 A, the maximum output voltage of ~ 75 V has been applied for the In NDs preparation in the patterned in-parallel cells. The electrolysis time was investigated, as demonstrated in Additional file 1: Figure S10. The ND arrays with highest density and uniformity were obtained at the reaction time of 5.0 min. Here, the optimized parameters of ITO film thickness of 25 nm, applied voltage of 75 V, and electrolysis reaction time of 5.0 min were employed for fabricating the large area SERS substrate with patterned ND arrays. The 50 × 50 μm^2^ square ND arrays with gap distance of about 5.0 μm have been achieved, as shown in Additional file 1: Figure S11.

**Fig. 6:**
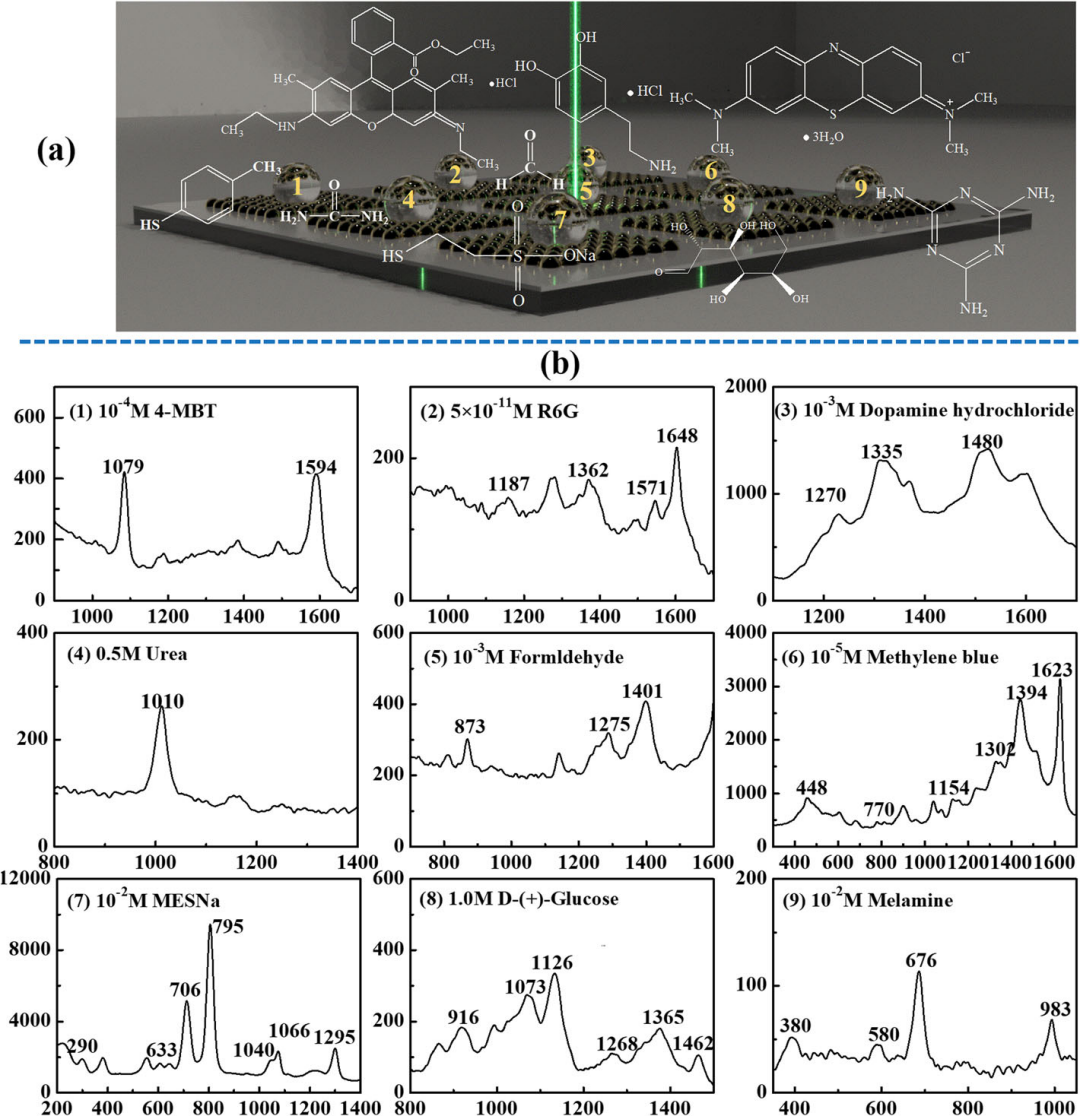
**a** Schematic of 9 sample droplets containing 9 different probe molecules being detected on one substrate with a 3 × 3 SERS arrays. **b** Raman spectra of the 9 probe molecules on each SERS area of the 3 × 3 arrays

A substrate with 3 × 3 areas prepared under the optimized conditions was used as a SERS substrate for multiple sample detection. As illustrated in Fig. 6a, 9 individual droplets (3.0 μL for each) containing 9 different analytic solutions were dripped at the patterned 9 areas. The selected 9 analytes 4-MBT [25], R6G [26], dopamine hydrochloride [34], urea [35], formaldehyde [36], methylene blue [37], MESNa [38, 39], d-(+)-Glucose [40], and melamine [41] were placed on the substrate as marked as samples 1 to 9, respectively. As seen from Fig. 6b, all 9 molecules show obvious Raman characteristic peaks. This has proven that the concept of using one substrate for simultaneously detection of various samples on one substrate.

## Conclusions

In summary, we have proposed and validated a simple, quick, and cheap method for fabricating NDs as SERS substrates on large-area surface with patternable structures. The formation of NDs was based on electrolysis of ITO film in water medium. The factors of electrolysis time, applied voltage, and ITO film thickness determined the ND size and density. Well-distributed NDs with size in the range of 50–60 nm have been obtained by electrolysis a 25-nm-thick ITO film at an applied voltage of 150 V for 1.5 min (droplet electrolysis). The fabricated ND substrate has been evaluated by its SERS effect after depositing ~ 77 nm Ag, using various probe molecules. Reproducible and sensitive Raman spectra have been obtained for 4-MBT and R6G with EFs of ~ 1.12 × 10^6^ and ~ 6.79 × 10^5^, respectively. Moreover, combined with photolithography, a 3.5 × 3.5 cm^2^ substrate could be patterned with 1, 9, or 25 SERS areas, for which multiple sample detection could be achieved simultaneously on one substrate with just one droplet of each analytic solution. This is highly required for quick qualification of specific molecules for on-site application situations like POCT, environmental monitoring, and airport security check. Such a technology shows advantages of easy fabrication under mild conditions, being patternable to form arrays on a large surface, and being integratable with microfluidics for high throughput optofluidic applications.

## Supplementary information

**Additional file 1:** **Figure S1.** EDS spectrum of ITO film. **Figure S2.** (a) SEM image of original FTO film. (b-e) SEM images of FTO film after electrolysis at different applied voltage 50, 100, 150 and 200 V, respectively. **Figure S3.** SEM images of different Ag film thicknesses deposited on NDs (a) 30 nm, (b) 77 nm, (c) 160 nm. (d) Raman spectra of 10^-4^ M 4-MBT on NDs deposited with different Ag film thicknesses. **Figure S4.** (a) Raman spectra of 10^-4^ M 4-MBT on NDs SERS substrate and pure powder of 4-MBT on glass substrate, (b) Raman spectra of 5 × 10^-7^ M R6G on NDs SERS substrate and pure powder of R6G on glass substrate. **Figure S5.** Schematic to estimate the number of probe molecules trapped in the "hot-spot" area (*N*_SERS_) among in the neighboring NDs. **Figure S6.** Raman spectra of 10^-3^ M MESNa on the NDs SERS substrate under the optimized conditions. **Figure S7.** Absorption spectrum of the NDs SERS substrate fabricated under optimized conditions. **Figure S8.** FDTD simulation of the electric field in the inter-gaps of NDs. **Figure S9.** Raman spectra of (a) 10^-4^ M 4-MBT and (b) 5 × 10^-7^ M R6G on ITO glass, NDs substrate after ITO electrolysis, ITO glass coated with 77 nm Ag film and NDs substrate coated with 77 nm Ag film, respectively. **Figure S10.** Fabrication of large area patterned ND arrays at different electrolysis time (a) 1.5 min, (b) 3.0 min, (c) 5.0 min, (d) 7.0 min, and (e) 10.0 min. **Figure S11.** SEM images of a patterned area after photolithography (a) before and (b) after electrolysis. SEM images of (c) the center and (d) the edge of a patterned ND area.

## Data Availability

All data generated or analyzed during this study are included in this article.

## Abbreviations

4-MBA: 4-Mercaptobenzoic
4-MBT: 4-Methylbenzenethiol
4-NBT: 4-Nitrobenzenethiol
CM: Chemical effect
DI: Deionized
EDS: Energy dispersive spectroscope
EF: Enhancement factor
EFs: Enhancement factors
EM: Electromagnetic effect
FDTD: Finite-difference time-domain
FE-SEM: Field emission-scanning electron microscopy
FTO: Fluorine-doped tin oxide
In: Indium
ITO: Indium tin oxide
KOH: Potassium hydroxide
LED: Light emission device
MESNa: Sodium 2-mercaptoethanesulfonate
ND: Nano-dot
POCT: Point-of-care technology
R6G: Rhodamine 6G
RSD: Relative standard deviation
SERS: Surface-enhanced Raman scattering
SERS: Surface-enhanced Raman spectroscopy
SPR: Surface plasmon resonance
TEM: Transmission electron microscopy
W: Wolfram
XRD: X-ray diffraction
