# Supplementary material for: Large-Area and Patternable Nano-Dot Array from Electrolysis of ITO Film for Surface-Enhanced Raman Spectroscopy
Source: Nanoscale Res Lett. 2020 Jan 13;15:8. doi: 10.1186/s11671-019-3239-9 (PMC6957589; doi:10.1186/s11671-019-3239-9)
Supplement: Supplementary file 1 — Additional file 1: Figure S1. EDS spectrum of ITO film. Figure S2. (a) SEM image of original FTO film. (b-e) SEM images of FTO film after electrolysis at different applied voltage 50, 100, 150 and 200 V, respectively. Figure S3. SEM images of different Ag film thicknesses deposited on NDs (a) 30 nm, (b) 77 nm, (c) 160 nm. (d) Raman spectra of 10-4 M 4-MBT on NDs deposited with different Ag film thicknesses. Figure S4. (a) Raman spectra of 10-4 M 4-MBT on NDs SERS substrate and pure powder of 4-MBT on glass substrate, (b) Raman spectra of 5 × 10^-7^ M R6G on NDs SERS substrate and pure powder of R6G on glass substrate. Figure S5. Schematic to estimate the number of probe molecules trapped in the "hot-spot" area (NSERS) among in the neighboring NDs. Figure S6. Raman spectra of 10-3 M MESNa on the NDs SERS substrate under the optimized conditions. Figure S7. Absorption spectrum of the NDs SERS substrate fabricated under optimized conditions. Figure S8. FDTD simulation of the electric field in the inter-gaps of NDs. Figure S9. Raman spectra of (a) 10-4 M 4-MBT and (b) 5 × 10^-7^ M R6G on ITO glass, NDs substrate after ITO electrolysis, ITO glass coated with 77 nm Ag film and NDs substrate coated with 77 nm Ag film, respectively. Figure S10. Fabrication of large area patterned ND arrays at different electrolysis time (a) 1.5 min, (b) 3.0 min, (c) 5.0 min, (d) 7.0 min, and (e) 10.0 min. Figure S11. SEM images of a patterned area after photolithography (a) before and (b) after electrolysis. SEM images of (c) the center and (d) the edge of a patterned ND area. [file 11671_2019_3239_MOESM1_ESM.docx]

**Supplementary Information**

**Large-area and Patternable Nano-Dot Array from Electrolysis of ITO Film for Surface-Enhanced Raman Spectroscopy**

Han Lu^1#^, Gengxin Han^1#^, Jieping Cao^1^, Mingliang Jin^1,2*^, Qilin Ma^3^, Eser Metin Akinogluc^2^, Xin Wang^1,2^, Li Nian^1^, Guofu Zhou^1,2^, and Lingling Shui^1,2,3*^

^1^ Guangdong Provincial Key Laboratory of Optical Information Materials and Technology, South China Academy of Advanced Optoelectronics, South China Normal University, Guangzhou 510006, China

^2^ International Academy of Optoelectronics at Zhaoqing, South China Normal University, Zhaoqing 526238, China

^3^ School of Information and Optoelectronic Science and Engineering, South China Normal University, Guangzhou 510006, China

E-mail: [shuill@m.scnu.edu.cn](mailto:shuill@m.scnu.edu.cn); [jinml@scnu.edu.cn](mailto:jinml@scnu.edu.cn)

1. **EDS spectrum of ITO film**


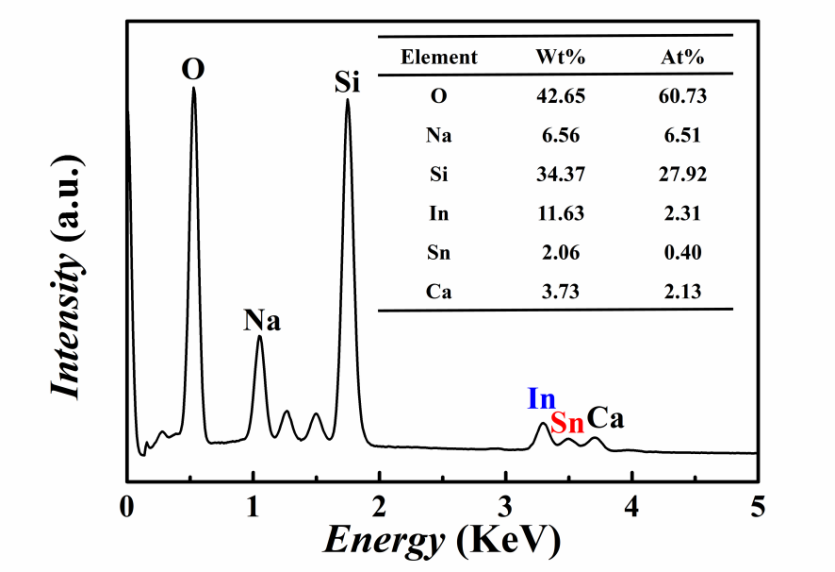


**Fig. S1.** EDS spectrum of ITO film.

1. **Electrolysis of FTO film**

Morover, we have also investigated the electrolysis of FTO glass, with the FTO thickness of 400 nm and square resistance of 10.85 Ω/sq. The electrolysis reaction was carried out for 1.5 min at different applied voltages. As shown in **Fig. S2**, micro- and nano- particles formed after electrolysis. This may suggest that such an electrolysis reaction is also applicable for other metal oxide films at various conditions and potential for other applications.


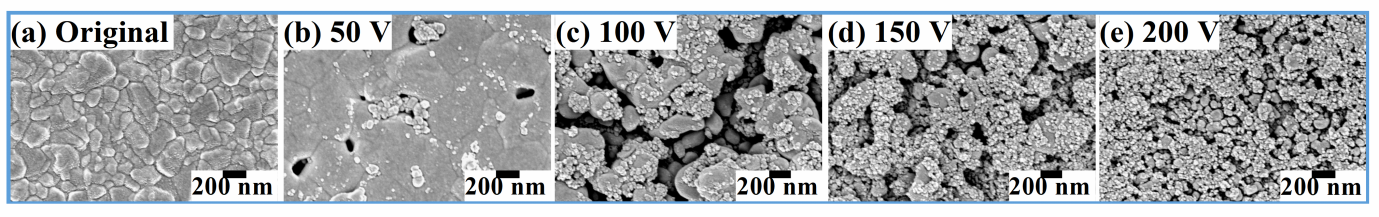


**Fig. S2.** (a) SEM image of original FTO film. (b-e) SEM images of FTO film after electrolysis for 1.5 min at different applied voltage 50, 100, 150 and 200 V, respectively.

1. **Effect of Ag film thickness**

Since the intensity of the Raman signal is mainly affected by the gap between the nanodots (NDs), the thickness of the metal coating is an important factor influencing the Raman enhancement. **Fig. S3(a-c)** show the SEM images of a series Ag film thickness of the deposited Ag film ranging from 30 nm to 160 nm. Raman enhancement is observed with the increase of the Ag film thickness from 30 nm to 77 nm, which is contributed to the gap between the NDs decrease, resulting in an increase of the local electromagnetic field of the NDs, and resulting in a significant increase in the intensity of the Raman signal (**Fig. S3d**). When the thickness of the Ag film increased from 77 nm to 160 nm where the gap are filled by the Ag film, Raman signal intensity decreases. The highest average Raman intensity was obtained at Ag thickness of 77 nm.





**Fig. S3.** SEM images of different Ag film thicknesses deposited on NDs (a) 30 nm, (b) 77 nm, (c) 160 nm. (d) Raman spectra of NDs deposited with different Ag film thicknesses using 10^-4^ M 4-MBT as a probe molecule.

1. **Calculation of the enhancement factor (EF)**

The experimental Raman EF of the SERS substrate was calculated by:

$$EF= \frac{I_{SERS}}{I_{bulk}}\frac{N_{bulk}}{N_{SERS}}$$

Where *I*_SERS_ denotes the Raman intensity of the 4-MBT or R6G molecules on the Ag deposited NDs SERS substrate and *I*_bulk_ represents the Raman intensity of the 4-MBT or R6G powder on glass slide. *N*_SERS_ and *N*_bulk_ are the number of the 4-MBT or R6G molecules contributed to the Raman spectra on the SERS substrate and on pure powder, respectively.


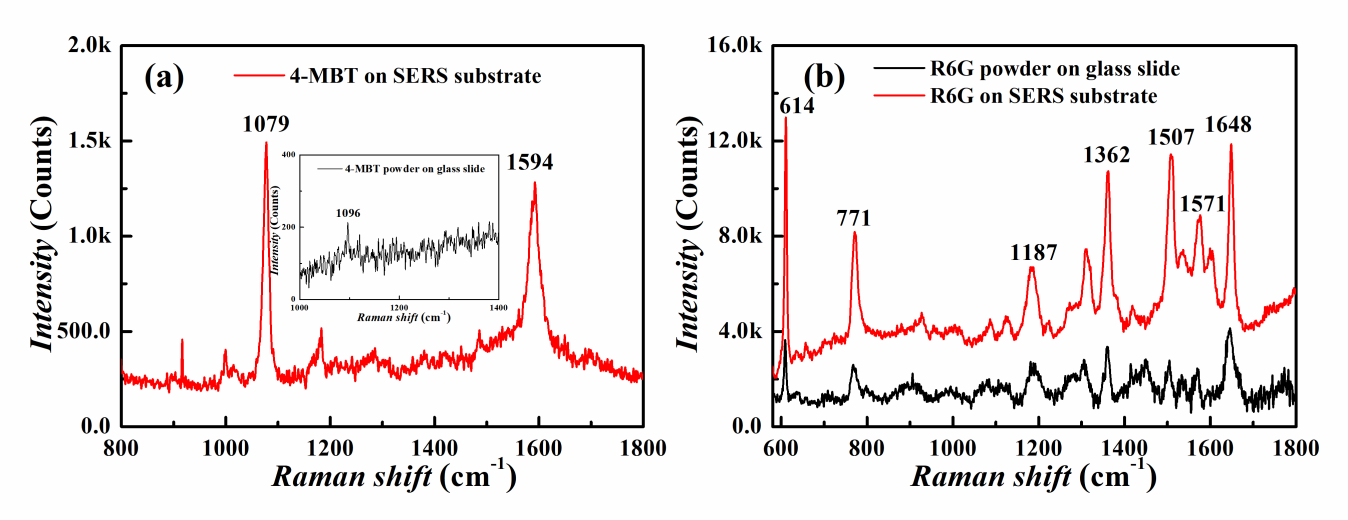


**Fig. S4.** (a) Raman spectra of 10^-4^ M 4-MBT on NDs SERS substrate (red curve) and pure powder of 4-MBT on glass substrate (black curve), (b) Raman spectra of 5×10^-7^ M R6G on NDs SERS substrate (red curve) and pure powder of R6G on glass substrate (black curve).

1. **EF for detecting 4-MBT**

*N*_bulk_ was determined by assuming that the laser excitation volume has a cylinder shape with the circular diameter being equal to the focused laser spot diameter and the height being equal to the effective probe depth (*H*_obj_ ). *H*_obj_ was obtained by adjusting the substrate stage out of the laser focus plane in 1 μm increments and capturing the silicon characteristic peak value at 520 cm^-1^. *N*_bulk_ is not counted when the signal intensity is less than half of the maximum value at the characteristic position, yielding the measured *H*_obj_ value is 26 μm. The amount of *N*_bulk_ that contributes to the pure powder Raman signal inside the interaction volume is calculated to be 1.76×10^11^ with the molar volume of 4-MBT 118.3 cm^3^ mol^-1^.

When determining *N*_SERS_, the number of the contributory molecules in the "hot-spot" region, the equation *N*_SERS_ = *A*_S_×*D*_4-MBT_ is applied, where *A*_S_ is the contributory "hot-spot" surface area and *D*_4-MBT_ is the surface density of the 4-MBT molecules chemisorbed on the metallic nanostructured surface [1, 2]. The model is employed to calculate *A*_S_, as shown in **Fig. S5**. In this calculation, *r* is the mean radius of NDs, and *h* is the gap distance which is assumed to be 5 nm for the NDs, assuming a 55 nm NDs and a 77 nm Ag film thickness. 4-MBT molecules are assumed to be absorbed as a monolayer with a surface density (*D*_4-MBT_) of 4 molecules/nm^2^ onto the surface area [3]. The diameter of the light spot in our Raman system is 1.30 μm, which includes approximately 344 "hot-spot" scattering sites contributed to the SERS signal enhancement based on the SEM images in **Fig. 3** in the main manuscript. *N*_SERS_ was obtained by multiplying the calculated total surface area of the 344 "hot-spots" region (*A*_S_ = 5.9×10^-13^ m^2^) by the surface density of the 4-MBT molecules chemisorbed on the nanogap surface (*D*_4-MBT_ = 4×10^18^ molecules/m^2^), yielding *N*_SERS_ = 2.36×10^6^. The EF of the "hot-spots" was calculated to be ~1.12×10^6^ at the Raman peak of 1079 cm^-1^.

**
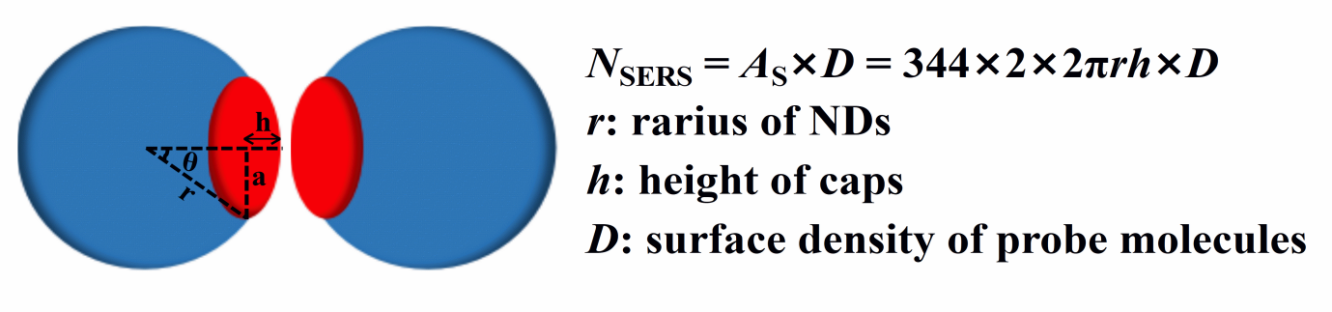
**

**Fig. S5.** Schematic to estimate the the number of probe molecules trapped in the "hot-spot" area (*N*_SERS_) among in the neighboring NDs. The "hot-spot" area is assumed to comprise a cap on the surface of each ND in the inter-particle region (red).

1. **EF for detecting R6G**

For the calculation of *N*_bulk_, the number of molecules *N*_bulk_ in the bulk sample inside the interaction volume (34.49 μm^3^) was calculated to be 5.68×10^10^ molecules. The average background intensity was subtracted from the Raman spectra to obtain the real peak value. The Raman intensity (*I*_bulk_) at 1648 cm^-1^ was calculated to 2240 counts.

To measure the number of *N*_SERS,_ the density of R6G molecules can be estimated to be sub-monolayer. Here the coverage of R6G molecules for a dense monolayer was estimated to be about 0.5 molecules/nm^2^ [4], the number of *N*_SERS_ was calculated to be 2.95×10^5^. Using identical conditions as for *I*_SERS_, Raman intensity at 1648 cm^-1^ were calculated to 7900 counts. The EF was ~6.79×10^5^ at the Raman peak of 1648 cm^-1^.

1. **SERS measurement of MESNa**

To further demonstrate the efficiency of the NDs SERS substrate, smaller Raman cross-section molecules of MESNa have also been for SERS measurement. **Fig. S6** shows the Raman spectra of 10^-3^ M MESNa. The major characteristic peaks are located at 290, 633, 706, 795, 1040, 1066 and 1295 cm^-1^. The peak at 290 cm^-1^ corresponds to the Ag-S stretching vibrations. The peaks at 633, 706 and 795 cm^-1^ were caused by C-S stretching vibration, the peaks at 633 and 706 cm^-1^ assigned to C-S (sulphur bound to the surface) stretching vibrations of a gauche and a trans conformer, respectively, the peak at 795 cm^-1^ represents sulphur from the sulfonic group. The peaks at 1040 and 1066 cm^-1^ can be assigned to the symmetric stretching vibration of the SO_3_^-^ group. The peak at 1295 cm^-1^ is due to the anti symmetric SO_3_^-^ stretch [5, 6].


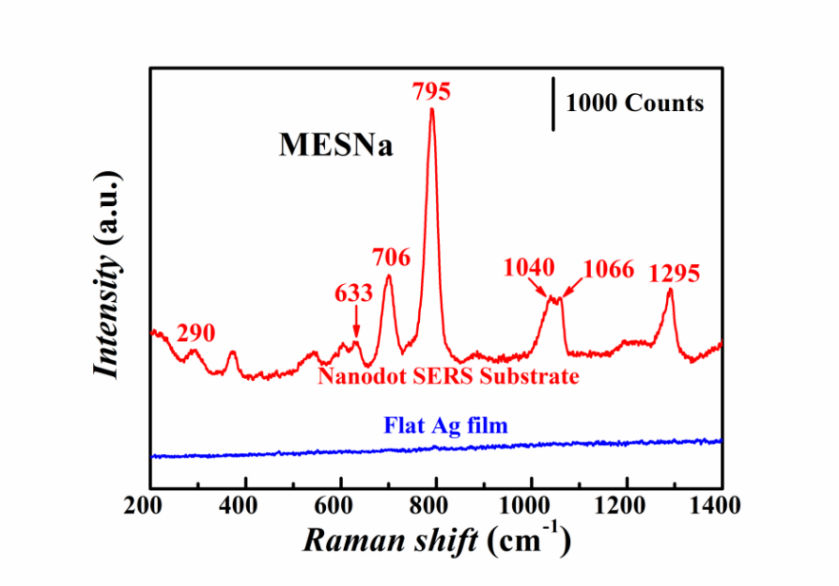


**Fig. S6.** Raman spectra of 10^-3^ M MESNa on the NDs SERS substrate under the optimized conditions. The reference substrate was prepared by depositing 77 nm Ag on a bare ITO (25 nm) film.

1. **Absorption spectrum of the NDs SERS substrate**


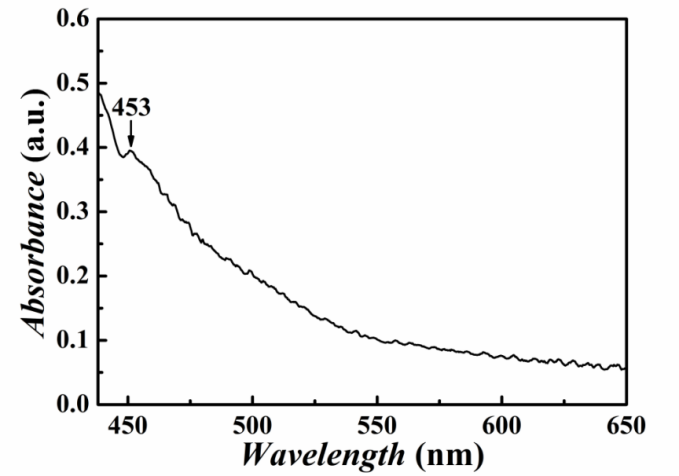


**Fig. S7.** Absorption spectrum of the NDs SERS substrate fabricated under optimized conditions.

1. **Finite-difference time-domain (FDTD) simulation**

The simulation was set up as a three-dimensional system with 0.5 nm resolution grid. The Ag-covered NDs and inter-gap of NPs used in these calculations are 100 and 5 nm, respectively, corresponding to the SEM image in **Fig. S3b**. The incident wavelength is 532 nm. **Fig. S8** shows the results of relative total electric field. Simulation results show the electric filed enhancement mainly occurs at the gaps between NDs. The maximum factor of 3.0 represents a field enhancement |E|^2^ of 10^3^ corresponds to a EF of 10^6^, which is in good agreement with the experimental results (1.12×10^6^ at 1079 cm^-1^ for 4-MBT and 6.79×10^5^ at 1648 cm^-1^ for R6G).


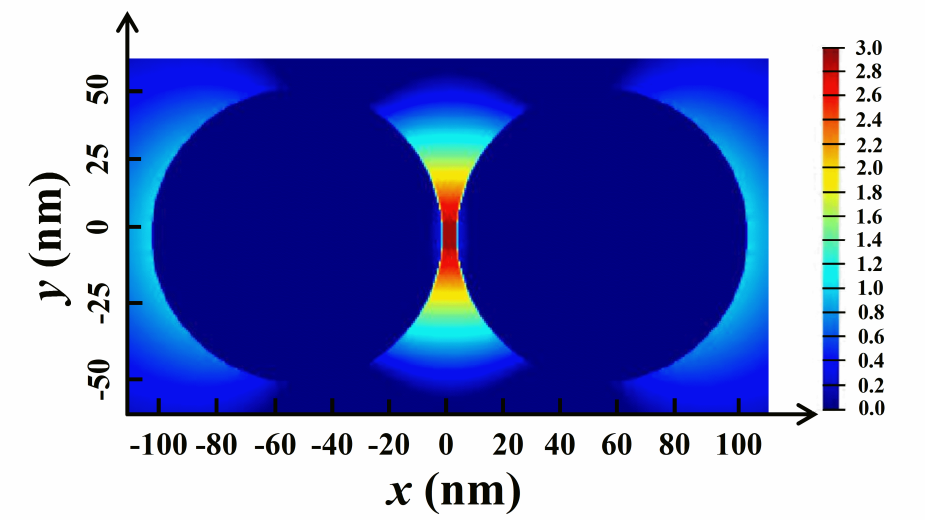


**Fig. S8.** FDTD simulation of the electric field in the inter-gaps of NDs.

1. **Raman spectra of 4-MBT and R6G**


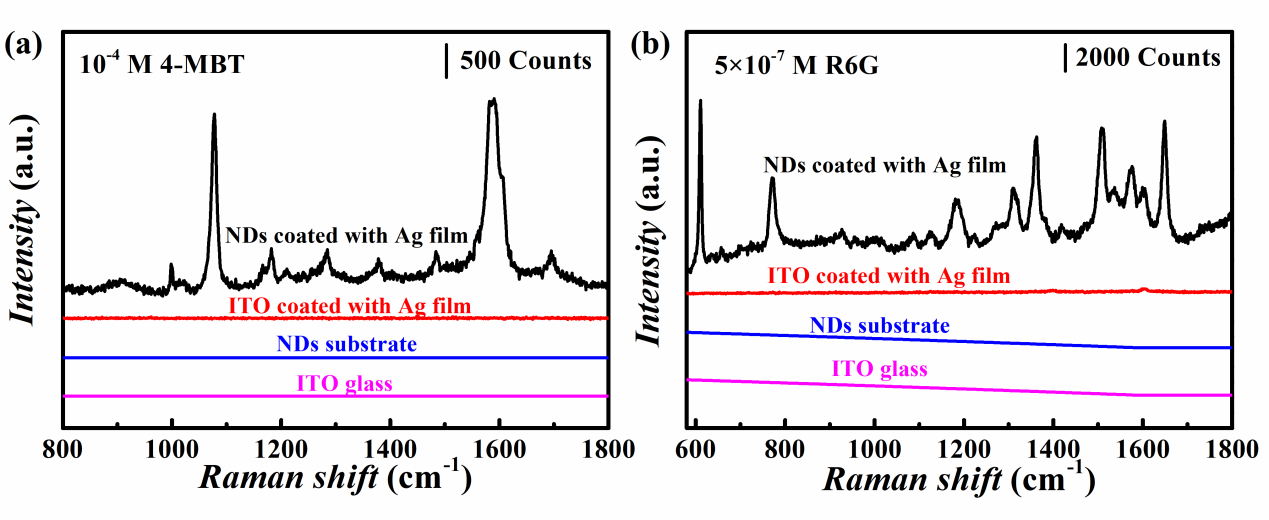


**Fig. S9.** (a) Raman spectra of 10^-4^ M 4-MBT on ITO glass, NDs substrate after ITO electrolysis, ITO glass coated with 77 nm Ag film and NDs substrate coated with 77 nm Ag film, respectively. (b) Raman spectra of 5×10^-7^ M R6G on ITO glass, NDs substrate after ITO electrolysis, ITO glass coated with 77 nm Ag film and NDs substrate coated with 77 nm Ag film, respectively.

1. **Optimized electrolysis time for fabrication patterned ND arrays**

In order to fabrication large area patterned ND arrays, a patterned and flat ITOs were bonded to form an in-parallel cell. Since the ITO area for electrolysis reaction was enhanced compared to the droplet electrolysis, the total charge number increases during electrolysis, inducing the increase in current and decrease in resistance. According to the current limit of the power supplier of 1.512 A, the maximum output voltage of ~75 V has been applied for the In NDs preparation in the patterned in-parallel cells. **Fig. S10** shows the SEM images of obtained NDs on the glass (ITO film thickness of 25 nm) at applied voltage of 75 V at different reaction time. The density of NDs increases and the gap between NDs decreases with the electrolysis time ranging from 1.5 to 5.0 min, as presented in **Figs. S10(a-c)**. When reaction time increases to 7.0 and 10.0 min, the size of obtained NDs increases, and the gap between NDs increases as well (**Fig. S10d and S10e)**, being similar to the droplet electrolysis (**Fig. 3a**). At the reaction time of 5.0 min, the uniformed and closed NDs are formed. Thus, 5.0 min is selected for fabrication large area patterned ND arrays SERS substrate.


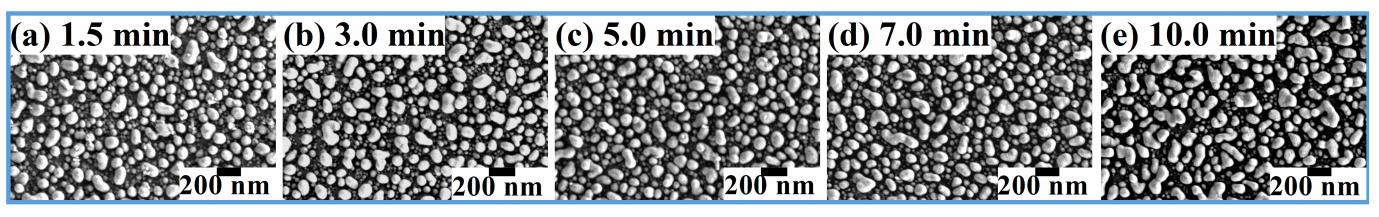


**Fig. S10**. Fabrication of large area patterned ND arrays at different electrolysis time (a) 1.5 min, (b) 3.0 min, (c) 5.0 min, (d) 7.0 min, and (e) 10.0 min, at a constant applied voltage of 75 V and ITO film thickness of 25 nm.

1. **SEM images of patterned ND array**

**
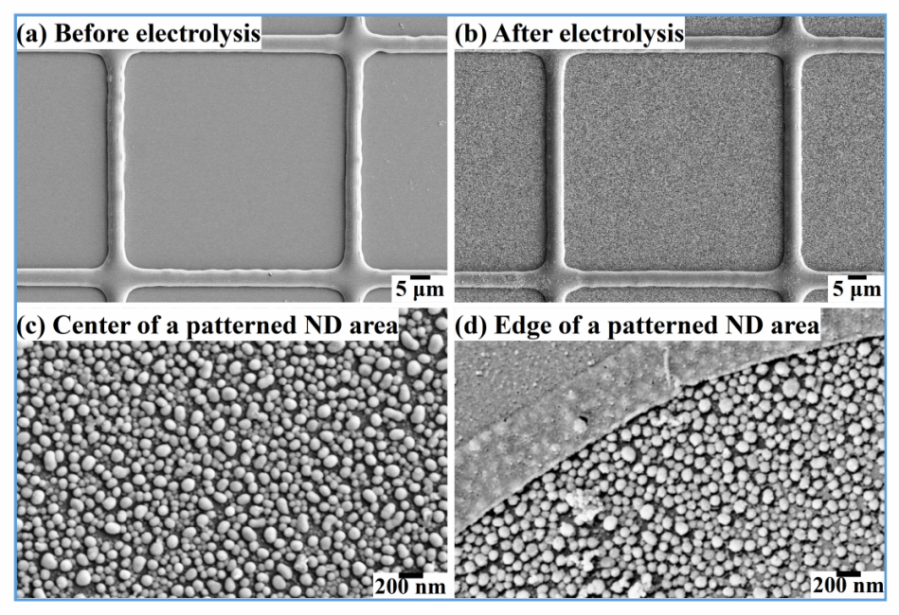
**

**Fig. S11**. SEM images of a patterned area after photolithography (a) before and (b) after electrolysis. SEM images of (c) the center and (d) the edge of a patterned ND area.

**References**

1. Li WY, Camargo PHC, Lu XM, Xia YN (2009) Dimers of silver nanospheres: facile synthesis and their use as hot spots for surface-enhanced Raman scattering. Nano Lett 9(1): 485-490

2. Wang J, Jin ML, Gong YX, Li H, Wu SJ, Zhang Z, Zhou GF, Shui LL, Eijkel JCT, van den Berg A (2017) Continuous fabrication of microcapsules with controllable metal covered nanoparticle arrays using droplet microfluidics for localized surface plasmon resonance. Lab Chip 17(11): 1970-1979

3. Zhang CT, Lin K, Huang YQ, Zhang J (2017) Graphene-Ag hybrids on laser-textured Si surface for SERS detection. Sensors-Basel 17(7): 1462

4.Liu LW, Zhou QW, Zeng ZQ, Jin ML, Zhou GF, Zhan RZ, Chen HJ, Gao XS, Lu XB, Senz S, Zhang Z, Liu JM (2016) Induced SERS activity in Ag@SiO2/Ag core-shell nanosphere arrays with tunable interior insulator. J Raman Spectrosc 47(10): 1200-1206

5. Piotrowski P, Bukowska J (2015) 2-Mercaptoethanesulfonate (MES) anion-functionalized silver nanoparticles as an efficient SERS-based sensor of metal cations. Sens Actuators B: Chem 221: 700-707

6. Chen Y, Wu LH, Chen YH, Bi N, Zheng X, Qi HB, Qin MH, Liao X, Zhang HQ, Tian Y (2012) Determination of mercury(II) by surface-enhanced Raman scattering spectroscopy based on thiol-functionalized silver nanoparticles. Microchim Acta 177(3-4): 341-348
